# The Relationship between Pulse Wave Velocity and Coronary Artery Stenosis and Percutaneous Coronary Intervention: a retrospective observational study

**DOI:** 10.1186/s12872-017-0476-7

**Published:** 2017-01-31

**Authors:** Hyung Joon Joo, Sang-A Cho, Jae-Young Cho, Jae Hyoung Park, Soon Jun Hong, Cheol Woong Yu, Do-Sun Lim

**Affiliations:** Department of Cardiology, Cardiovascular Center, Korea University Anam Hospital, 126-1, 5ka, Anam-dong, Sungbuk-ku, Seoul, 136-705 Republic of Korea

**Keywords:** Aortic stiffness, Pulse wave velocity, Coronary artery stenosis, Percutaneous coronary artery intervention

## Abstract

**Background:**

Arterial stiffness has been suggested as a valuable predictor of coronary artery stenosis (CAS). However, little data are available on aortic stiffness and CAS in patients who have previously undergone percutaneous coronary artery intervention (PCI). The aim of this study was to investigate the association of arterial stiffness to CAS in patients with a history of PCI and those without a history of PCI.

**Methods:**

We retrospectively studied 1093 consecutive patients who had undergone coronary angiography (CAG). Arterial stiffness was determined by brachial-ankle pulse wave velocity (baPWV) measured prior to CAG.

**Results:**

In patients without a history of PCI, baPWV significantly increased in patients with CAS compared to that in patients without CAS (*p* < 0.001). However, among patients with a history of PCI, there was no significant difference in baPWV. Multivariate logistic regression analysis demonstrated that baPWV was an independent risk predictor for CAS in patients without a history of PCI, but not in those with a history of PCI (OR 1.106, 95% CI 1.039–1.177, *p* = 0.002). In CAS patients without a history of PCI, increased baPWV was significantly associated with multiple cardiovascular risk factors, multivessel involvement, and anatomical severity.

**Conclusions:**

Prediction of CAS by baPWV is significantly attenuated in patients with a history of PCI.

**Electronic supplementary material:**

The online version of this article (doi:10.1186/s12872-017-0476-7) contains supplementary material, which is available to authorized users.

## Study highlights

1. baPWV is significantly increased in CAS patients without a history of PCI compared to those non-CAS patients.
2. baPWV is an independent risk predictor for CAS
3. Higher baPWV is associated with multiple vessel involvement and angiographic severe disease.
4. The relationship between baPWV and CAS was attenuated in patients with abnormal ABI.

## Background

Despite advances in risk stratification and its treatment, coronary artery disease remains a leading cause of morbidity and mortality worldwide. Coronary atherosclerosis and subsequent coronary plaque development are the main pathognomonic signs of coronary artery disease. Thus, early detection of coronary plaque and coronary artery stenosis (CAS) is important for the management of this disease.

Coronary angiography (CAG) continues to be considered the gold standard procedure for the diagnosis of CAS. However, its invasiveness limits its clinical use exclusively to subjects with a high-risk profile for CAS. Although many non-invasive techniques may be used to detect CAS, including the treadmill test, stress echocardiography, myocardial perfusion scanning, and computed tomographic angiography, these too have limitations when applied to asymptomatic subjects or subjects with a low-risk profile.

Recently, arterial stiffness has emerged as a potential candidate for the prediction of cardiovascular disease [1–3]. It too is associated with other cardiovascular risk factors, including age, hypertension and diabetes mellitus [4–6]. Various methods have been employed to measure arterial stiffness. Among them, pulse wave velocity (PWV) and the augmentation index (AIx) have been frequently used as reliable indicators of arterial stiffness [7]. Brachial-ankle pulse wave velocity (baPWV) can be measured non-invasively to estimate the conventional carotid-femoral PWV [8]. However, the association of baPWV or AIx with CAS is still controversial. Some studies have reported that baPWV is a meaningful risk predictor for CAS [9, 10]. In contrast, other studies have reported the limited value of baPWV to predict CAS [11]. Moreover, the discrepancy of baPWV and AIx for CAS has been also reported [12, 13]. At present, no data are available on baPWV and AIx in patients who have previously undergone percutaneous coronary intervention (PCI).

The purpose of the present study was to explore the association between arterial stiffness (measured by PWV and AIx) and CAS in patients who have already undergone percutaneous coronary intervention (PCI) as compared to that in patients without a history of PCI treatment.

## Methods

### Study design

**Fig. 1:**
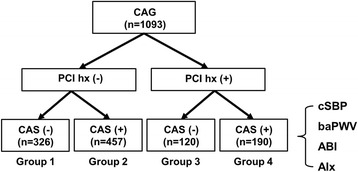
Study design. CAG: coronary angiography, PCI: percutaneous coronary angiography, CAS: coronary artery stenosis, cSBP: central systolic blood pressure, baPWV: brachial-ankle pulsed wave velocity, ABI: ankle-brachial index, AIx: augmentation index

This was a single-center, retrospective study of consecutive patients who underwent both CAG and arterial stiffness measurements from January 2011 through January 2013. It was approved by the ethical committee of the institutional review board of Korea University Anam Hospital, and the need for written informed consent was waived due to the non-interventional and retrospective nature of the study. After excluding the patients with acute coronary syndrome, underlying valvular heart disease or prior surgical coronary revascularization, a total of 1093 patients were identified and divided into 4 groups as follows: (Group 1, *n* = 326) absence of CAS without prior PCI history, (Group 2, *n* = 457) presence of CAS without prior PCI history, (Group 3, *n* = 120) absence of CAS with prior PCI history, and (Group 4, *n* = 190) presence of CAS with prior PCI history (Fig. 1).

### Coronary artery stenosis (CAS)

CAS was angiographically defined as a >50% diameter stenosis in at least one major coronary artery (left anterior descending artery, left circumflex artery, or right coronary artery). The extent of vessel involvement was divided into 0-, 1-, 2-, and 3-vessel groups. The anatomical severity of CAS was determined by using the modified Gensini score [14].

### Non-invasive hemodynamic parameter measurement

baPWV and ankle-brachial index (ABI) were measured non-invasively using an oscillometric sphygmomanometric device (VP-1000 plus; Omron Colin, Kyoto, Japan). Central systolic blood pressure (cSBP) and AIx were also measured non-invasively by using an applanation tonometry device (HEM9000A1; Omron Colin, Kyoto, Japan). cSBP was estimated from the late systolic shoulder (SBP2) of the radial pulse waveform. Because AIx is significantly influenced by heart rate, an index normalized for a heart rate of 75 bpm (AIx@75) was used. All these measurements were performed the day prior to CAG. The validity and reproducibility of the measurements have been previously described [8, 15].

### Statistical analysis

Continuous variables were expressed as the mean ± SD. Comparisons between the 2 groups were performed using an independent Student’s *t*-test for continuous variables and *χ* ^2^ test for categorical variables. To identify the risk predictors for CAS, selected factors from all variables were tested using a multivariate logistic regression analysis by univariate analysis (*p* < 0.10). An odds ratio with a 95% confidence interval and *P*-value were reported. Receiver operating characteristic (ROC) curves were constructed for the diagnosis of significant coronary artery stenosis. Two academic authors separately analyzed the database and reconciled any discrepancies. All tests were two-sided. A *P*-value of less than 0.05 was considered statistically significant. All statistical analyses were performed using the Statistical Package for the Social Sciences (SPSS) software package (Version 18.0, SPSS Inc., Chicago, IL, USA).

## Results

### Baseline demographic characteristics

Demographic features of study subjects are presented in Table 1. Patients with a history of PCI (Groups 3 and 4) were significantly older, predominantly male, and presented with a higher incidence of typical or atypical chest pain, hypertension, diabetes, dyslipidemia, and impaired renal function compared to the patients without a history of PCI (Groups 1 and 2). Patients with a history of PCI were more frequently receiving ACE inhibitors, angiotensin receptor blockers, beta-blockers, calcium channel blockers, insulin and statins than patients with no history of PCI. However, among the patients without a history of PCI, the patients with CAS (Group 2) had more typical chest pain compared to those without CAS (Group 1). Among patients with a history of PCI, patients without CAS (Group 3) were older and had a higher incidence of diabetes than patients with CAS (Group 4).

**Table 1:** Baseline demographic characteristics

Variables	Group 1	Group 2	*p*-value (1 vs 2)	Group 3	Group 4	*p*-value (3 vs 4)	*p*-value (1/2 vs 3/4)
Age (year)	58.9 ± 10.04	59 ± 10.89	0.896	63.88 ± 8.32	61.41 ± 10.04	0.020	<0.001
Male (%)	182 (55.83%)	259 (56.67%)	0.814	84 (70%)	148 (77.89%)	0.119	<0.001
Symptoms							
Asymptomatic	70 (20.5%)	66 (14.4%)	0.001	75 (62.5%)	96 (50.5%)	0.078	<0.001
Atypical chest pain	127 (39.0%)	149 (32.6%)		15 (12.5%)	24 (12.6%)		
Typical chest pain	129 (39.6%)	242 (53.0%)		30 (25.0%)	70 (36.8%)		
BMI (kg/m2)	24.9 ± 3.09	24.71 ± 2.97	0.391	24.68 ± 2.8	24.99 ± 3.04	0.356	0.683
Smoking (%)	97 (29.75%)	138 (30.2%)	0.894	38 (31.67%)	51 (26.84%)	0.360	0.671
Hypertension (%)	191 (58.4%)	266 (58.3%)	1.000	108 (90.0%)	180 (94.7%)	0.172	<0.001
Diabetes mellitus (%)	61 (18.71%)	82 (17.94%)	0.784	46 (38.33%)	50 (26.32%)	0.026	<0.001
Dyslipidemia (%)	95 (29.14%)	149 (32.6%)	0.302	102 (85%)	164 (86.32%)	0.746	<0.001
CKD (%)	69 (21.23%)	110 (24.07%)	0.352	42 (35%)	53 (27.89%)	0.186	0.008
Uric acid (mg/dL)	5.14 ± 1.48	5.25 ± 1.44	0.337	5.3 ± 1.63	5.42 ± 1.34	0.493	0.085
Hba1c (% of THb)	6.19 ± 1.04	6.29 ± 1.41	0.539	6.48 ± 1.08	6.02 ± 0.82	0.005	0.644
TC (mg/dL)	147.8 ± 89.48	157.3 ± 103.2	0.168	123.7 ± 63.19	133.7 ± 80.2	0.226	<0.001
LDL-C (mg/dL)	96.5 ± 26.88	96.13 ± 27.24	0.853	72.64 ± 20.31	73.07 ± 21.8	0.860	<0.001
HDL-C (mg/dL)	51.31 ± 13.57	49.95 ± 12.83	0.157	48.1 ± 11.29	46.89 ± 11.36	0.361	<0.001
Triglyceride (mg/dL)	137.5 ± 89.21	152.3 ± 105.9	0.036	119.6 ± 67.36	129.9 ± 82.59	0.231	<0.001
Creatinine (mg/dL)	0.96 ± 0.46	0.95 ± 0.23	0.672	1.06 ± 0.65	1.02 ± 0.2	0.479	0.003
hsCRP (mg/L)	2.74 ± 8.99	2.15 ± 5.68	0.324	2.01 ± 4.32	1.82 ± 4	0.701	0.162
#, risk factors^a^	2.23 ± 1.39	2.29 ± 1.35	0.579	3.67 ± 1.25	3.48 ± 0.97	0.152	<0.001
Index Medications							
ACE inhibitors (%)	12 (3.68%)	15 (3.28%)	0.763	35 (29.17%)	55 (28.95%)	0.967	<0.001
ARBs (%)	87 (26.69%)	101 (22.1%)	0.139	52 (43.33%)	68 (35.79%)	0.184	<0.001
Beta-blockers (%)	35 (10.74%)	62 (13.57%)	0.236	47 (39.17%)	88 (46.32%)	0.216	<0.001
CCB (%)	84 (25.77%)	135 (29.54%)	0.246	47 (39.17%)	83 (43.68%)	0.432	<0.001
Diuretics (%)	27 (8.28%)	28 (6.13%)	0.245	12 (10%)	15 (7.89%)	0.522	0.340
Insulin (%)	2 (0.61%)	2 (0.44%)	1.000	4 (3.33%)	5 (2.63%)	0.739	0.003
Statins (%)	77 (23.62%)	116 (25.38%)	0.573	102 (85%)	163 (85.79%)	0.848	<0.001

*BMI* body mass index, *CKD* chronic kidney disease, *TC* total cholesterol, *LDL*-*C* low-density lipoprotein cholesterol, *HDL*-*C* high-density lipoprotein cholesterol, *ACE* angiotensin-converting enzyme, *ARB* angiotensin receptor blocker, *CCB* calcium channel blocker

^a^Risk factors included old age (≥65 years), male gender, smoking, hypertension, diabetes mellitus, and dyslipidemia. Data are presented as means ± SDs for continuous variables and numbers (%) for categorical variables

### Non-invasive hemodynamic parameters and CAS

Non-invasive hemodynamic parameters are summarized in Table 2. Among patients without a history of PCI, those with CAS (Group 2) had a significantly lower diastolic blood pressure (DBP), higher pulse pressure, lower cSBP, higher baPWV, and lower ABI than patients without CAS (Group 1). However, among patients with a history of PCI, those with CAS (Group 4) had a significantly higher pulse pressure than those without CAS (Group 3).

**Table 2:** Comparisons of SBP, DBP, pulse pressure, cSBP, baPWV, ABI, Aix, and AIx@75

Variables	Group 1	Group 2	*p*-value (1 vs 2)	Group 3	Group 4	*p*-value (3 vs 4)	*p*-value (1/2 vs 3/4)
SBP (mmHg)	130.3 ± 17.72	130.7 ± 17.12	0.762	129.2 ± 15.15	131.9 ± 17.25	0.196	0.804
DBP (mmHg)	77.56 ± 10.77	74.12 ± 11.76	<0.001	78.34 ± 9.98	76.26 ± 11.8	0.141	0.067
Pulse pressure (mmHg)	52.75 ± 13.95	56.6 ± 13.37	<0.001	50.84 ± 12.74	55.63 ± 13.45	0.004	0.222
cSBP (mmHg)	135.2 ± 20.99	134.2 ± 19.33	0.529	134.5 ± 18.23	136.3 ± 19.71	0.467	0.488
baPWV (right, cm/sec)	1518.6 ± 292.1	1607.4 ± 342.6	<0.001	1521.4 ± 241.3	1572.4 ± 339.3	0.124	0.409
baPWV (left, cm/sec)	1517.4 ± 292.0	1592.4 ± 335.5	0.001	1533.0 ± 250.9	1559.6 ± 319.0	0.416	0.574
baPWV (average, cm/sec)	1517.7 ± 289.1	1599.8 ± 326.7	<0.001	1526.8 ± 241.6	1566.0 ± 323.8	0.226	0.476
ABI (right)	1.13 ± 0.09	1.12 ± 0.1	0.142	1.12 ± 0.08	1.12 ± 0.11	0.795	0.343
ABI (left)	1.13 ± 0.08	1.1 ± 0.12	<0.001	1.12 ± 0.08	1.11 ± 0.11	0.571	0.604
ABI (average)	1.13 ± 0.07	1.11 ± 0.1	0.007	1.12 ± 0.08	1.12 ± 0.11	0.700	0.820
AIx (%)	81.73 ± 14.64	80.96 ± 14.36	0.492	82.19 ± 16.25	81.37 ± 13.8	0.661	0.700
AIx@75 (%)	79.8 ± 13.26	78.11 ± 12.5	0.090	80.58 ± 14.02	79.23 ± 11.81	0.402	0.314

Data are presented as means ± SDs

*SBP* systolic blood pressure, *DBP* diastolic blood pressure, *cSBP* central systolic blood pressure, *baPWV* brachial-ankle pulsed wave velocity, *ABI* ankle-brachial index, *AIx* augmentation index, *AIx*@*75* AIx normalized for a heart rate of 75 bpm

To explore the potential risk predictors for CAS, we performed univariate and multivariate logistic regression analyses (Table 3). In the total population, the use of RAS inhibitors and statins, DBP, cSBP, baPWV, ABI, and AIx@75 were independent risk predictors for CAS. In patients without a history of PCI, hypertension, the use of CCB, DBP, cSBP, baPWV, ABI, and AIx@75 were independent risk predictors for CAS. In patients with a history of PCI, diabetes mellitus, DBP, cSBP, and AIx@75 were independent risk predictors for CAS.

**Table 3:** Univariate and multivariate analyses for coronary artery stenosis (CAS)

Variable	Univariate analysis			Multivariate analysis		
	Exp(B)	OR (95% CI)	*p*-value	Exp(B)	OR (95% CI)	*p*-value
Total population						
Age	0.995	0.983–1.007	0.403			
Male	1.148	0.896–1.470	0.276			
BMI	0.995	0.956–1.036	0.808			
Smoking	0.951	0.730–1.237	0.706			
Hypertension	0.840	0.659–1.071	0.159			
Diabetes mellitus	0.812	0.608–1.085	0.159			
Dyslipidemia	1.184	0.930–1.509	0.171			
CKD	1.013	0.767–1.339	0.926			
RAS inhibitor	0.843	0.658–1.080	0.177	0.680	0.497–0.931	0.016
Beta-blocker	1.340	0.991–1.811	0.057			
CCB	1.222	0.941–1.586	0.132			
Statin	1.131	0.885–1.445	0.325	1.477	1.084–2.014	0.014
SBP	1.004	0.996–1.011	0.356			
DBP	0.977	0.965–0.988	<0.001	0.951	0.935–0.967	<0.001
PP	1.023	1.013–1.034	<0.001			
cSBP	0.999	0.993–1.006	0.876	1.021	1.008–1.033	0.001
baPWV	1.001	1.000–1.001	<0.001	1.075	1.021–1.133	0.006
ABI	0.202	0.053–0.774	0.020	0.094	0.019–0.468	0.004
AIx@75	0.990	0.980–1.001	0.064	0.976	0.963–0.989	<0.001
Patients without prior PCI history						
Age	1.001	0.987–1.014	0.895			
Male	1.035	0.777–1.378	0.814			
BMI	0.980	0.935–1.027	0.390			
Smoking	1.021	0.749–1.393	0.894			
Hypertension	0.796	0.599–1.059	0.118	0.667	0.462–0.964	0.031
Diabetes mellitus	0.950	0.658–1.371	0.783			
Dyslipidemia	1.176	0.864–1.602	0.303			
CKD	1.176	0.836–1.655	0.352			
RAS inhibitor	0.806	0.586–1.108	0.183			
Beta-blocker	1.305	0.839–2.029	0.237			
CCB	1.208	0.878–1.662	0.247	1.552	1.032–2.333	0.035
Statin	1.100	0.790–1.532	0.573			
SBP	1.001	0.993–1.010	0.762			
DBP	0.974	0.961–0.987	<0.001	0.952	0.933–0.971	<0.001
PP	1.022	1.010–1.034	<0.001			
cSBP	0.998	0.990–1.005	0.528	1.016	1.001–1.030	0.034
baPWV	1.001	1.000–1.001	<0.001	1.106	1.039–1.177	0.002
ABI	0.123	0.024–0.621	0.011	0.039	0.006–0.273	0.001
AIx@75	0.990	0.978–1.002	0.091	0.979	0.963–0.995	0.010
Patients with prior PCI history						
Age	0.972	0.948–0.997	0.026			
Male	1.510	0.898–2.539	0.120			
BMI	1.038	0.959–1.123	0.355			
Smoking	0.792	0.480–1.307	0.361			
Hypertension	0.928	0.578–1.488	0.756			
Diabetes mellitus	0.575	0.352–0.937	0.027	0.556	0.318–0.970	0.039
Dyslipidemia	1.113	0.581–2.132	0.747			
CKD	0.718	0.440–1.174	0.187			
RAS inhibitor	0.735	0.450–1.198	0.217			
Beta-blocker	1.340	0.842–2.132	0.217			
CCB	1.205	0.757–1.919	0.433			
Statin	1.066	0.559–2.033	0.847			
SBP	1.010	0.995–1.026	0.196			
DBP	0.983	0.961–1.006	0.142	0.953	0.923–0.984	0.003
PP	1.029	1.008–1.049	0.005			
cSBP	1.005	0.992–1.018	0.465	1.032	1.010–1.054	0.004
baPWV	1.000	1.000–1.001	0.256			
ABI	0.640	0.058–7.095	0.716			
AIx@75	0.992	0.972–1.011	0.401	0.971	0.947–0.996	0.024

RAS inhibitor refers to ACE inhibitors or ARBs

*CKD* chronic kidney disease, *OR* odds ratio, *95*% *CI* 95% confidence interval

Thus, baPWV significantly increased in CAS patients without a history of PCI as an independent risk predictor for CAS.

### baPWV and coronary artery disease risk factors

**Fig. 2:**
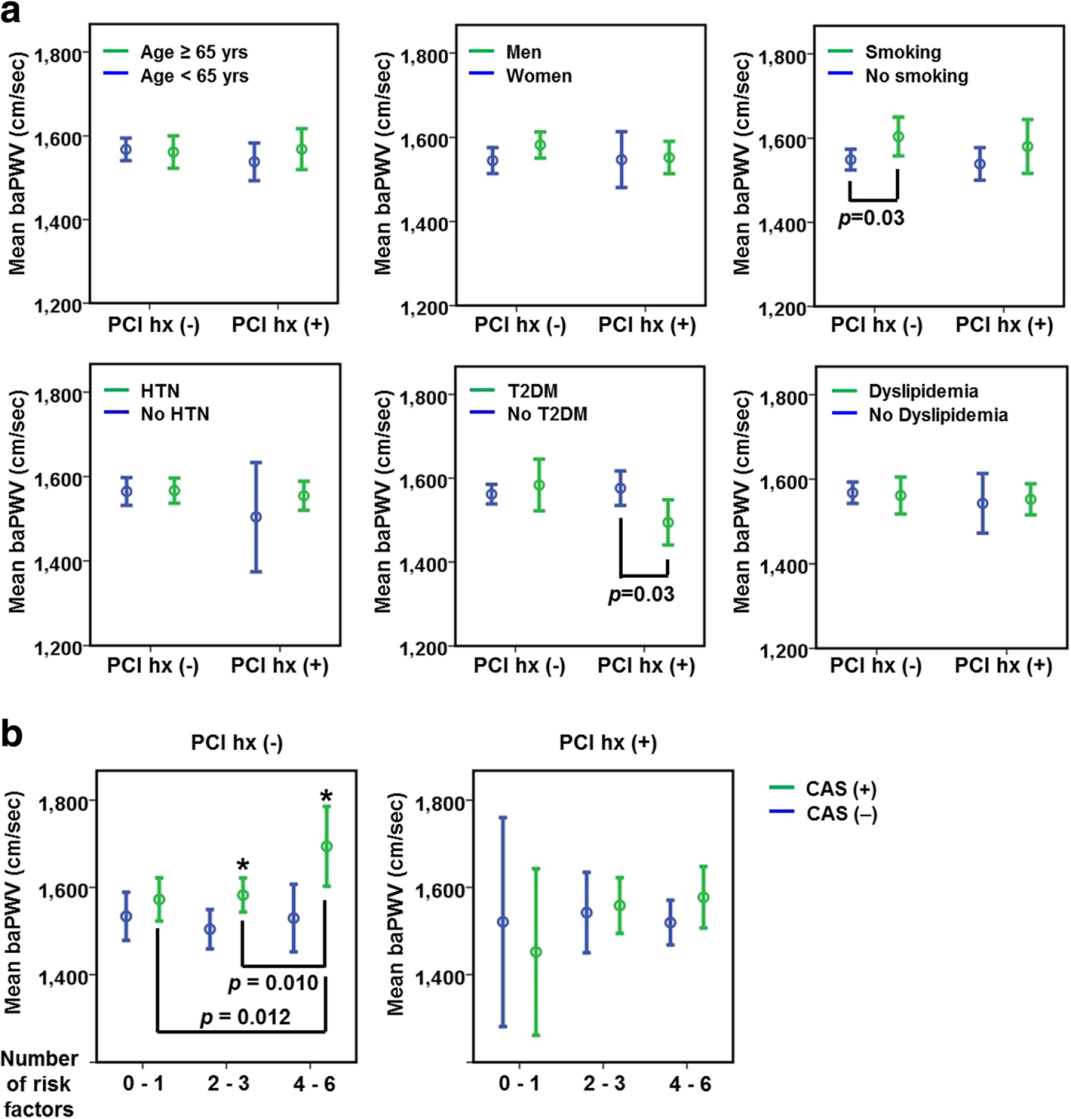
baPWV and coronary artery disease risk factors. **a** Comparisons of baPWV according to coronary artery disease risk factors and history of PCI. **b** Comparisons of baPWV according to CAS and the number of coronary artery disease risk factors. *p < 0.05 versus CAS (−). HTN: hypertension, T2DM: type 2 diabetes mellitus

Next, we compared baPWVs dependent to the presence or absence of conventional cardiovascular risk factors, which included age (≥65 years), gender, smoking, hypertension, type 2 diabetes mellitus, and dyslipidemia (Fig. 2a). Among patients without a history of PCI, baPWV increased significantly in smokers than in non-smokers (*p* = 0.03). baPWV was also higher in male patients than female patients, although this difference was not significant (*p* = 0.10). Among patients with a history of PCI, baPWV decreased significantly in diabetic patients than in non-diabetic patients (*p* = 0.03). Interestingly, among patients without a history of PCI, baPWV increased significantly in patients with CAS or multiple conventional coronary artery disease risk factors (Fig. 2b). However, among patients without a history of PCI, the number of conventional coronary artery risk factors or CAS did not affect baPWV. These data suggest that increased baPWV in CAS patients without a history of PCI could be associated with an overall increase in cardiovascular risk.

### baPWV and the severity of CAS

**Fig. 3:**
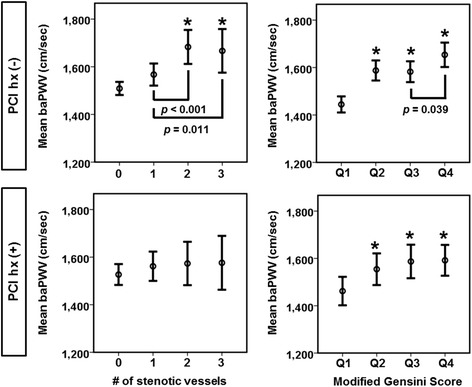
baPWV and angiographical disease severity. Comparisons of baPWV according to the number of stenotic coronary arteries or the modified Gensini score. *p < 0.05 versus patients with no stenotic coronary artery or the lowest modified Gensini score (Q1)

Although baPWV did not significantly differ with the numbers of stenotic major coronary arteries in patients with a history of PCI, baPWV increased significantly in cases of 2-vessel or greater involvement in patients without a history of PCI (Fig. 3, *left panel*). Patients were divided into 4 groups according to their modified Gensini score: Q1 = zero, Q2 = 0.5 to 9.5, Q3 = 10 to 29.5 and Q4 = 30 and over. In both patients with and without a history of PCI, baPWVs in Q2, Q3, and Q4 were significantly higher than those in Q1 (Fig. 3, *right panel*). This suggests that baPWV increased significantly even in patients with mild coronary artery stenosis (the modified Gensini score > 0.5), regardless of a history of PCI. Interestingly, in patients without a history of PCI, baPWV in Q4 was significantly higher than that in Q3 (*p* = 0.039). When taken in conjunction with the extent of vessel involvement, these data suggest that baPWV showed a trend towards a positive correlation with anatomical severity and multivessel involvement in patients without a history of PCI.

### Effect of ABI on baPWV and CAS

**Fig. 4:**
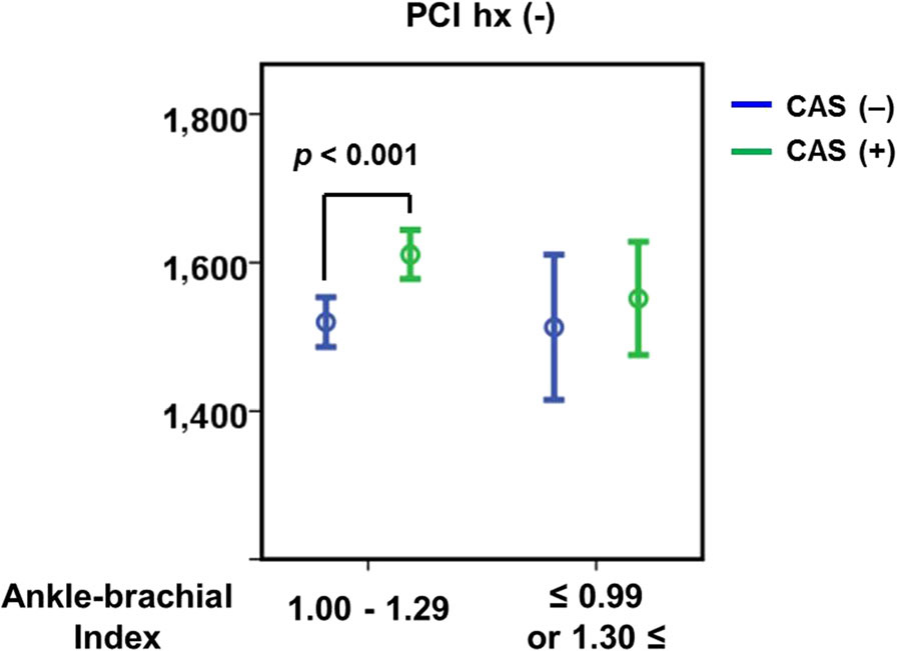
Comparisons of baPWV according to ABI and CAS in patients without a history of PCI

ABI is a well-known indicator for peripheral artery disease [16]. The reference value for ABI is known to be 1.0–1.29. A value of less than 0.9 suggests significant stenosis of the lower limb artery. A value greater than 1.3 suggests non-compressible calcified vessels. An abnormal value of ABI could indicate atherosclerosis or an arteriosclerotic change to the blood vessels [17]. In the present study, we further analyzed baPWV and CAS dependence on ABI. Interestingly, in patients without a history of PCI, baPWV significantly increased only in CAS patients with normal ABI (Fig. 4). Abnormal ABI attenuated the baPWV increase in CAS patients. This suggests that baPWV could not predict CAS in patients with vascular atherosclerosis or arteriosclerotic changes as determined by ABI.

### Diagnostic potential of baPWV for CAS

**Fig. 5:**
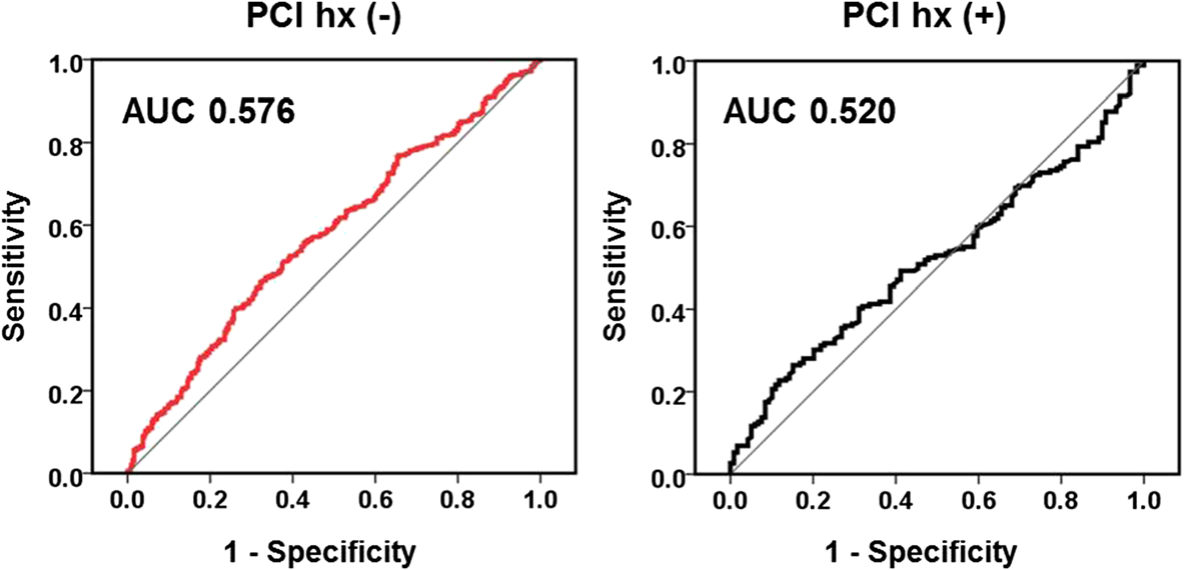
ROC curves of baPWV in patients with or without a history of PCI

Based on the ROC curves, we analyzed the accuracy of baPWV in predicting CAS. ROC curves in patients with and without a history of PCI are presented in Fig. 5. The area under the curve (AUC) of baPWV in patients with a history of PCI was determined to be non-significant (AUC = 0.520, *p* = 0.548), while that of baPWV in patients without a history of PCI was significantly higher than the reference value (AUC = 0.576, *p* < 0.001). Using a cut-off value of 1488.5 cm/s for baPWV, a sensitivity of 59.6% and specificity of 50.2% were obtained. A cut-off value of 1561 cm/s for baPWV produced a 50.8% sensitivity and a 62.5% specificity value.

## Discussion

Coronary atherosclerosis and arteriosclerosis are the pathognomonic processes leading to coronary vascular injury. Arterial stiffness reflects the functional and structural vascular changes that occur during atherosclerosis and arteriosclerosis [18]. Previous studies have demonstrated that arterial stiffness is significantly associated with an increased risk of coronary artery disease [2, 19, 20] and cardiovascular events [21, 22]. Moreover, measurement of arterial stiffness is often recommended to evaluate vascular damage in daily clinical practice. Recent guidelines have recommended carotid-femoral PWV measurement for the evaluation of asymptomatic organ damage in hypertensive patients [23]. Although carotid-femoral PWV has been used as a conventional method for the measurement of arterial stiffness, baPWV has been extensively studied as an alternative method given its ease and convenience.

Numerous studies have shown that an increased baPWV is significantly associated with the presence of CAS [9–11]. In the present study, baPWV significantly increased only in CAS patients without a history of PCI (Table 2). In addition, multivariate analyses showed baPWV to be an independent predictor for CAS only in patients without a history of PCI (Table 3). Considering that previous studies in the literature excluded patients with a history of PCI, the results of the present study correspond to those previously published.

Formerly, arterial stiffness was known to be associated with conventional cardiovascular risk factors, including age, hypertension, and diabetes mellitus [4–6]. The findings of the present study show that, although there were significant differences in clinical profiles (including cardiovascular risk factors) between patients with and without a history of PCI, the presence of each conventional cardiovascular risk factor (age, gender, smoking, hypertension, diabetes mellitus, and dyslipidemia) had little effect on baPWV in both patients with and without a history of PCI (Fig. 2a). Interestingly, baPWV increased in CAS patients without a history of PCI while overall cardiovascular risk increased (Fig. 2b). This association between baPWV and overall cardiovascular risk was not shown in patients without CAS or patients with a history of PCI. These findings suggests that baPWV could play a role in predicting CAS in patients at modest cardiovascular risk, such as those with more than 2 cardiovascular risk factors and no history of PCI.

In previous studies, the relationship between baPWV and the severity of CAS have shown conflicting results. Xiong et al. reported a strong relationship between baPWV and CAS severity as assessed by the SYNTAX score [24]. On the other hand, Chae et al. reported a non-significant relationship between baPWV and multivessel involvement of CAS [9]. Both studies included only patients without a history of PCI. Interestingly, the present study demonstrated that an increased baPWV was related to both multivessel involvement and the increased, modified Gensini score in patients without a history of PCI. Furthermore, this relationship was attenuated in patients with a history of PCI.

In this study, baPWV was not increased in CAS patients with a history of PCI. There are several factors to consider when explaining the relationship between baPWV and CAS and a history of PCI as observed in the present study.

First, the baseline demographic characteristics showed significant differences between the patients with and without a history of PCI (Table 1). Patients with a history of PCI were older, male-predominant and frequently asymptomatic. These patients were also presented with an increased incidence of hypertension, diabetes, dyslipidemia and renal function impairment. The medication which was administered to these patients varied widely. Previously published studies have reported different results regarding this relationship between baPWV and CAS in different study populations. Seo et al. reported that baPWV had the limited value as a predictor of CAS in the patients with a high-risk profile [25]. Park et al. also showed that baPWV failed to predict CAS in the patients with suspected stable angina by means of a standard medical history and stress test [26]. When we analyzed the diagnostic potential of baPWV according to the pre-test probability based on the patient symptom, baPWV was significant for CAS only in asymptomatic or atypical chest pain patients without prior PCI history (Additional file 1: Table S1). It supported the previous underlying assumption that baPWV could have potential to predict CAS in low probability patients.

Second, atherosclerosis and arteriosclerosis in other types of arteries could affect baPWV and act as confounding factors in the prediction of CAS. While both conditions are known to elicit overall vascular changes as opposed to localized effects, they often cause different types of vascular diseases such as coronary artery disease and other peripheral occlusive arterial disease. ABI is thought to be reflective of atherosclerotic changes to the peripheral arteries in lower limbs [27, 28] and is widely-used to evaluate and diagnose peripheral artery disease [29–31]. Conventionally, a low ABI (<0.9) represents significant arterial stenosis of the lower extremities and a high ABI (>1.2) represents abnormal arterial stiffness and hardening. Interestingly, although there was no significant difference in baPWV according to ABI and CAS in patients with a history of PCI in this study (data not shown), comparison of baPWV according to ABI in patients without a history of PCI did show that baPWV was elevated only in CAS patients with normal ABI (Fig. 4). This further suggests that vascular changes in other arteries may have significantly limited the efficacy of baPWV in the prediction of CAS, even in patients without a history of PCI.

Finally, the present study showed that the overall diagnostic potential of baPWV for CAS, even in patients without a history of PCI, was somewhat limited. Although the AUC in the ROC curve was statistically significant, a cut-off value of 1561 cm/s produced only a 50.8% sensitivity and a 62.5% specificity (Fig. 5). Based on previous reports, the suggested cut-off values of baPWV for the prediction of CAS vary greatly from 1540 cm/s to 2150 cm/s [9, 10]. Sensitivities and specificities for CAS in the literature are 65.0–76.7% and 56.7–61.0%, respectively, which are too low to have any clinical implications. In addition, previous studies have also shown that baPWV failed to predict the risk of revascularization [9].

The limitations of this study must be taken into consideration. First, this study was a single-center, retrospective, cross-sectional study, which cannot determine causality. Second, we did not assess the treatment decisions made, including revascularization for patients with CAS. Third, although the present study successfully showed the significance of baPWV for CAS, its diagnostic potential was quite poor. To investigate its clinical implication, it should be kept being researched whether baPWV could provide the additional statistical power to the conventional patient stratification, and finally improve the overall predictability for CAS.

## Conclusions

In conclusion, baPWV could play a role in predicting CAS and its severity only in patients without a history of PCI. However, its diagnostic potential for CAS is quite limited, and vascular changes in other arteries detected by ABI significantly attenuate their correlation. The clinical value of follow-up baPWV measurement in patients with CAS remains to be further investigated.

## Additional file

Additional file 1: Table S1. ROC curve analysis for baPWV to predict CAS. (DOCX 13 kb)

## Abbreviations

ABI: Ankle-brachial index
AIx: Augmentation index
AUC: Area under the curve
baPWV: Brachial-ankle pulse wave velocity
CAG: Coronary angiography
CAS: Coronary artery stenosis
cSBP: Central systolic blood pressure
DBP: Diastolic blood pressure
PCI: Percutaneous coronary artery intervention
PWV: Pulse wave velocity
ROC: Receiver operating characteristic
