# Supplementary material for: The Relationship between Pulse Wave Velocity and Coronary Artery Stenosis and Percutaneous Coronary Intervention: a retrospective observational study
Source: BMC Cardiovasc Disord. 2017 Jan 31;17:45. doi: 10.1186/s12872-017-0476-7 (PMC5282734; doi:10.1186/s12872-017-0476-7)
Supplement: Additional file 1: Table S1. — ROC curve analysis for baPWV to predict CAS. (DOCX 13 kb) [file 12872_2017_476_MOESM1_ESM.docx]

**Table S1. ROC curve analysis for baPWV to predict CAS**

|  | **AUC (95% confidence interval)** |
| --- | --- |
| **Total patients** |  |
| **Asymptomatic + atypical chest pain (n=622)** | **0.575 (0.530-0.620)** |
| Asymptomatic (n=307) | 0.576 (0.511-0.640) |
| Atypical chest pain (n=315) | 0.574 (0.510-0.637) |
| **Typical chest pain (n=471)** | **0.541 (0.487–0.595)** |
| **Patients without prior PCI history** |  |
| **Asymptomatic + atypical chest pain (n=412)** | **0.595 (0.540-0.650)** |
| Asymptomatic (n=136) | 0.628 (0.534-0.722) |
| Atypical chest pain (n=276) | 0.575 (0.507-0.643) |
| **Typical chest pain (n=371)** | **0.556 (0.495-0.617)** |
| **Patients with prior PCI history** |  |
| **Asymptomatic + atypical chest pain (n=210)** | **0.536 (0.458-0.615)** |
| Asymptomatic (n=171) | 0.530 (0.442-0.617) |
| Atypical chest pain (n=39) | 0.569 (0.378-0.761) |
| **Typical chest pain (n=100)** | **0.519 (0.402-0.636)** |
